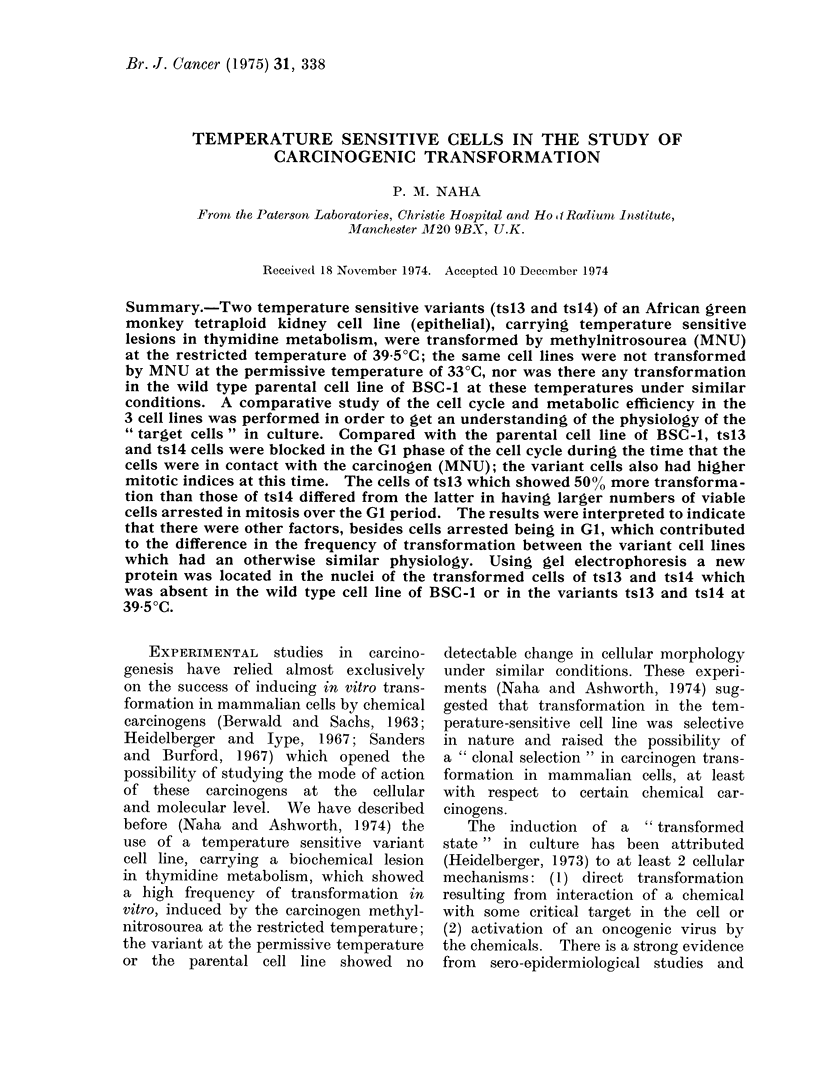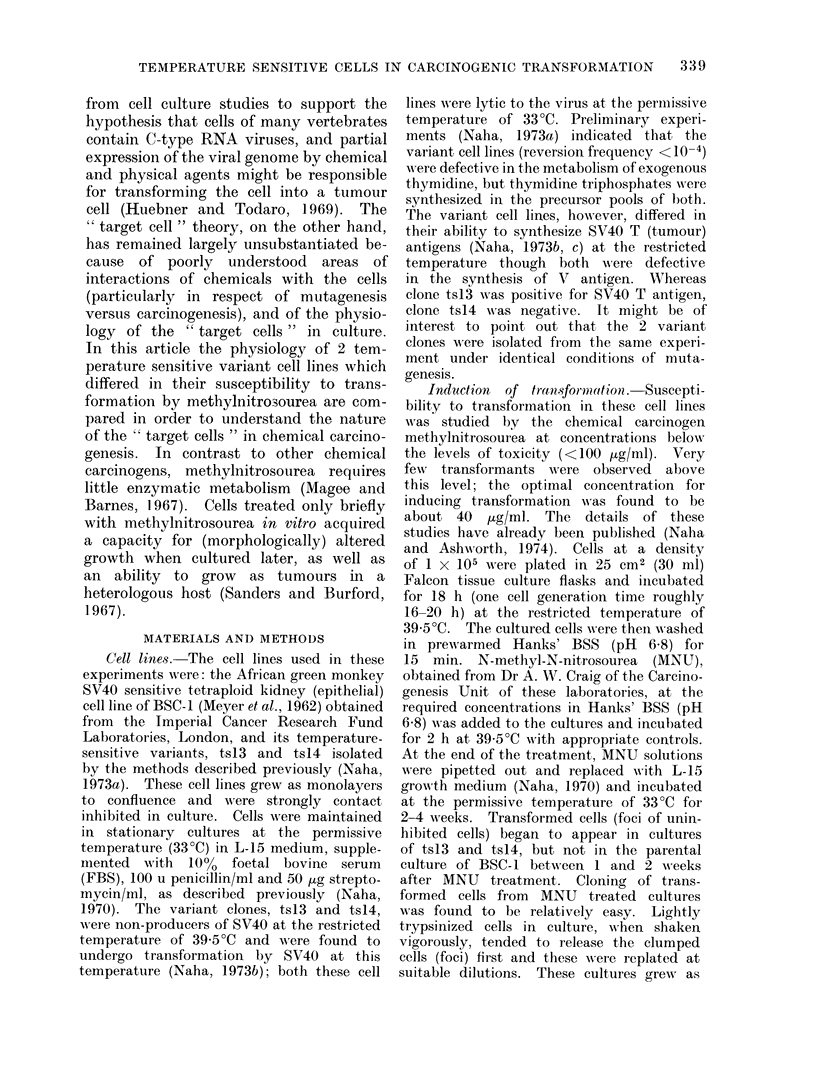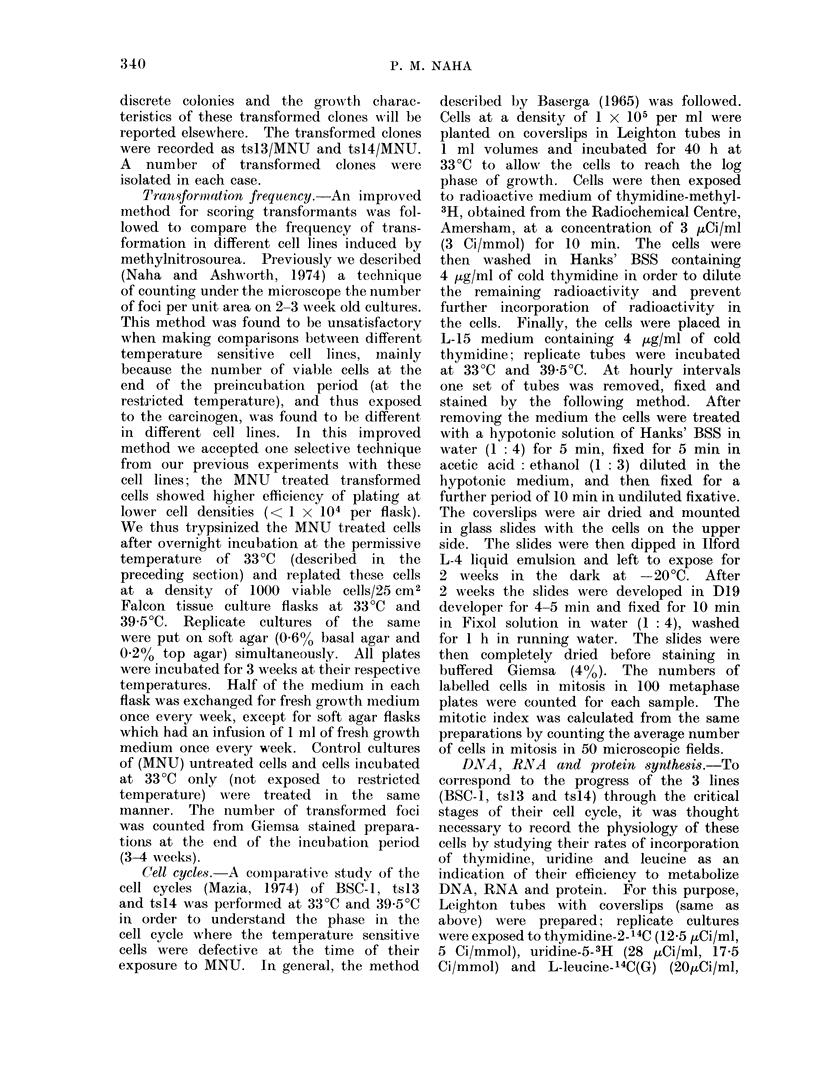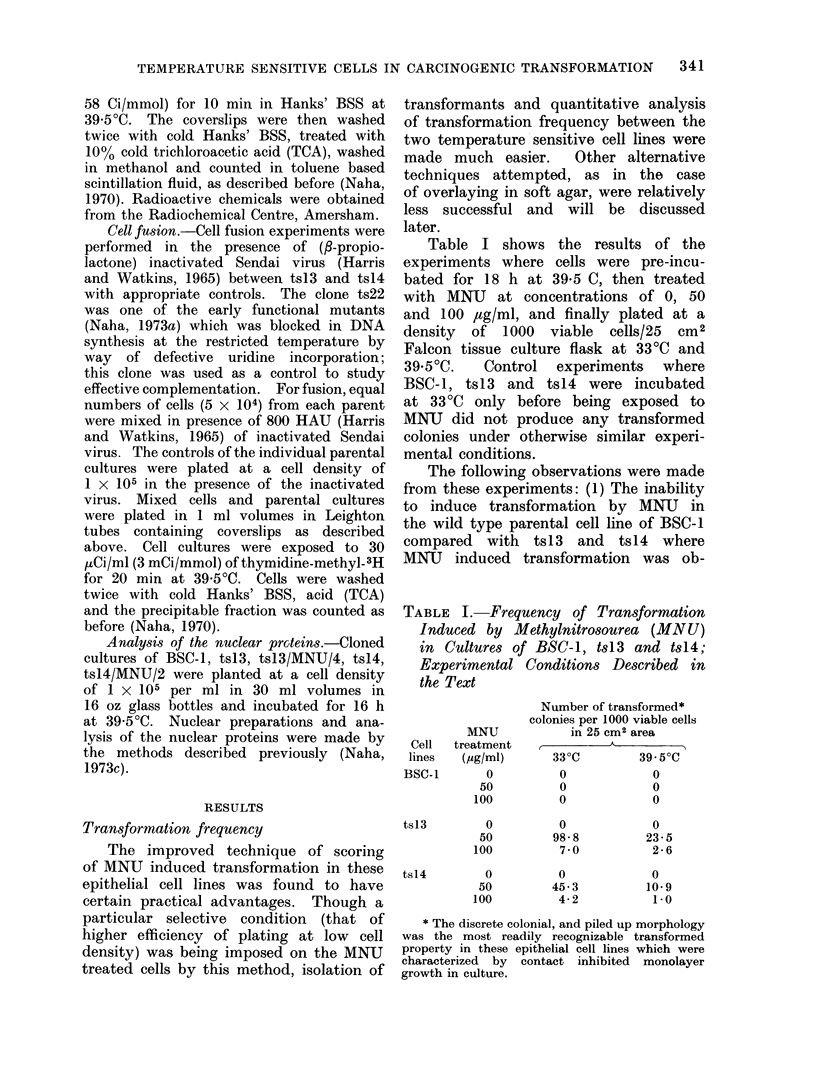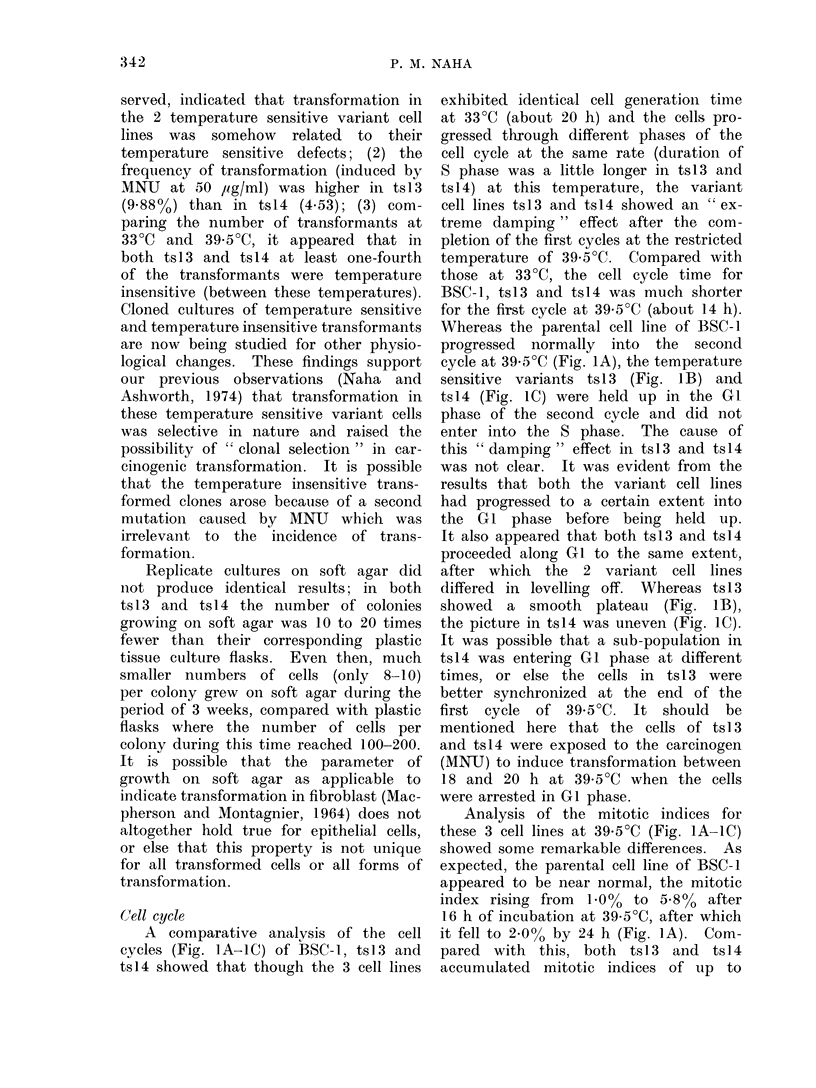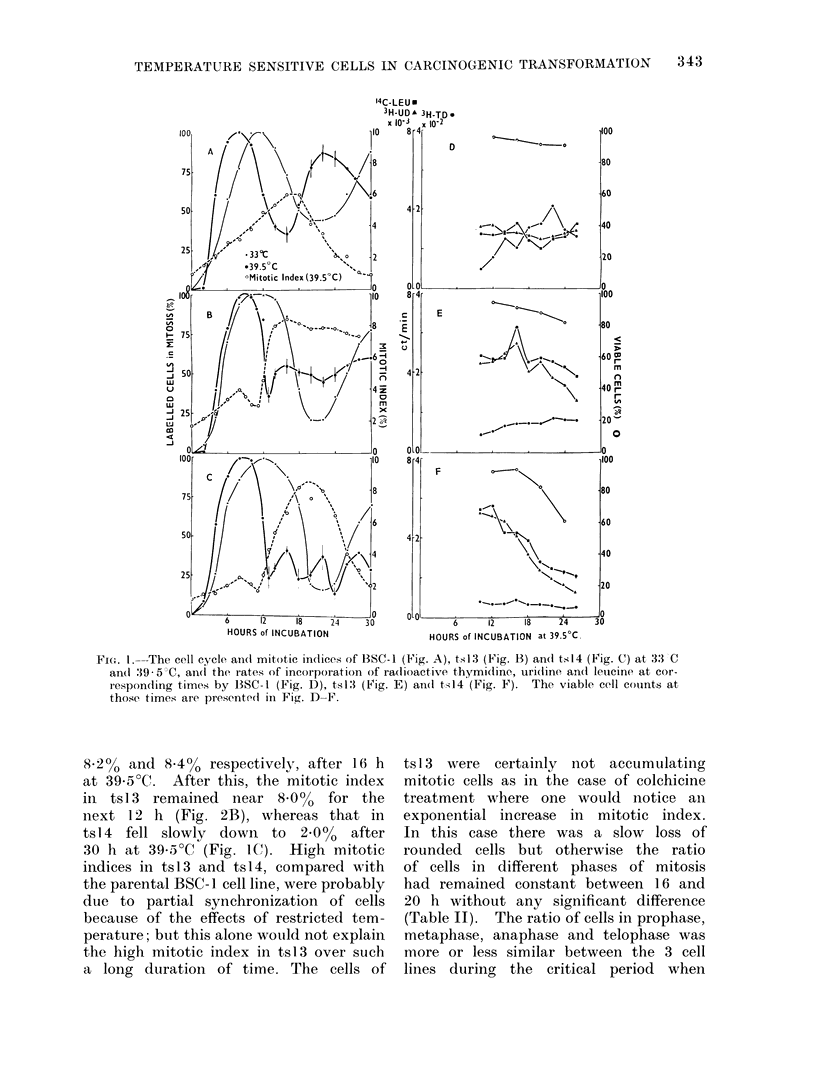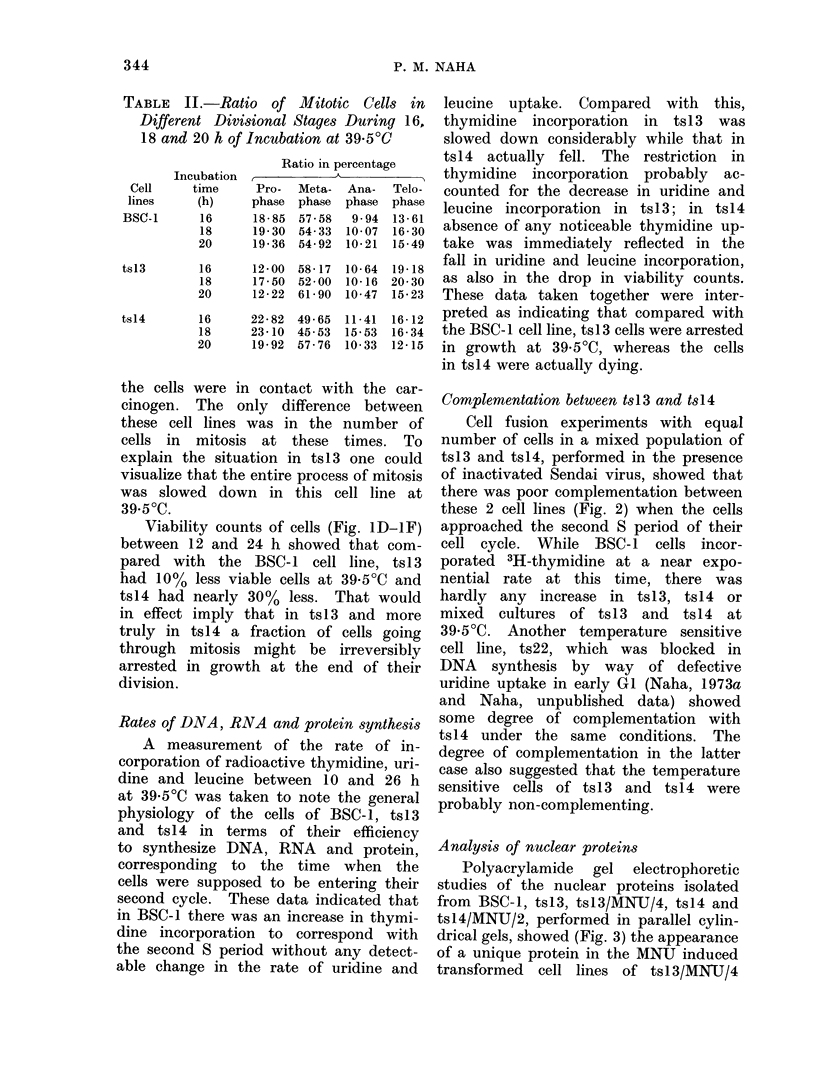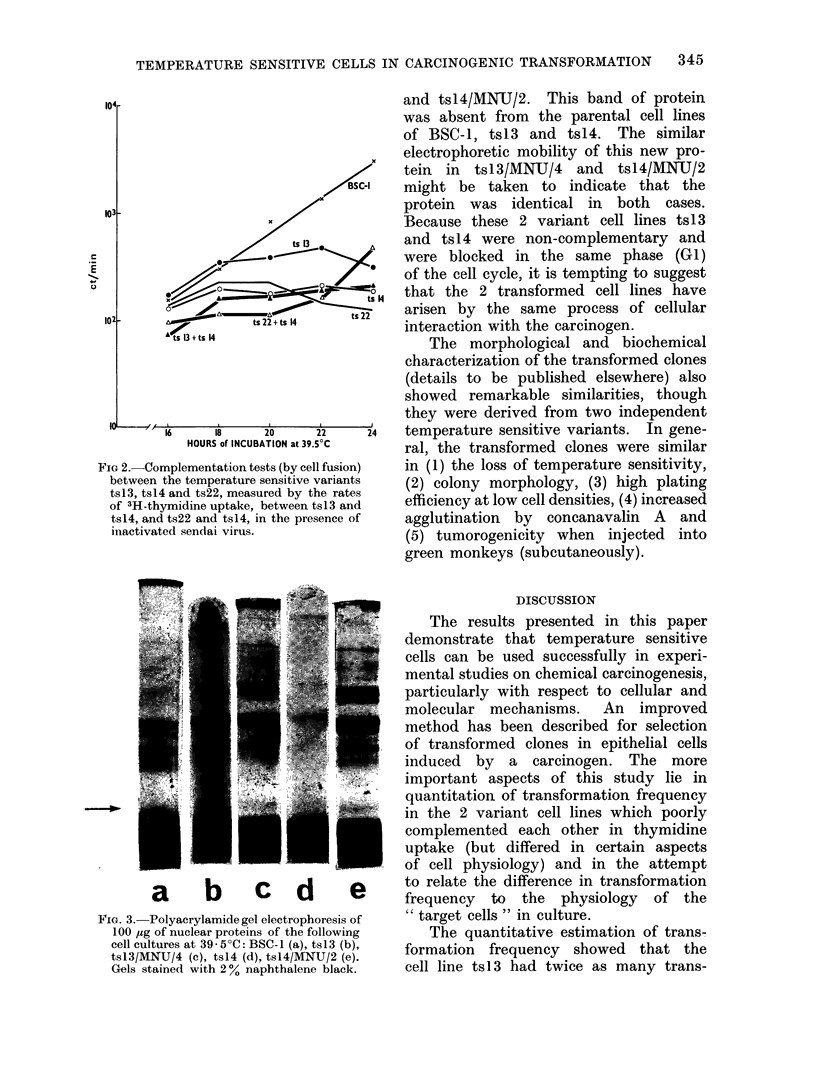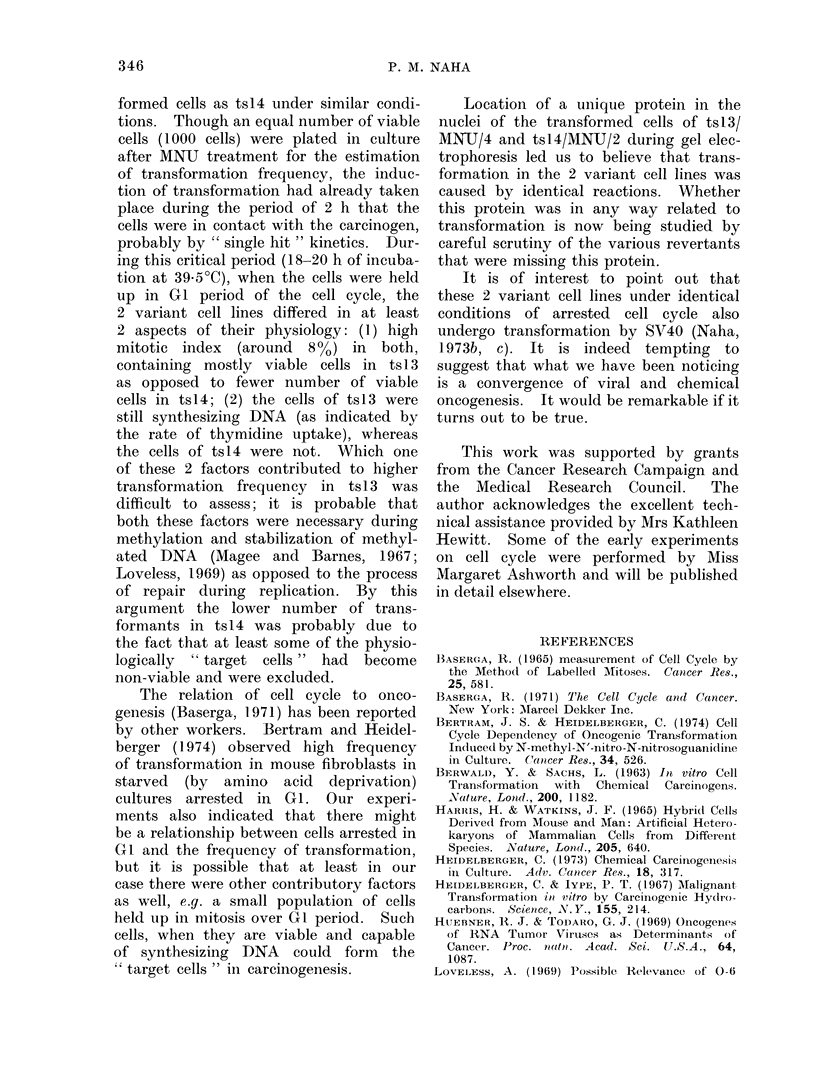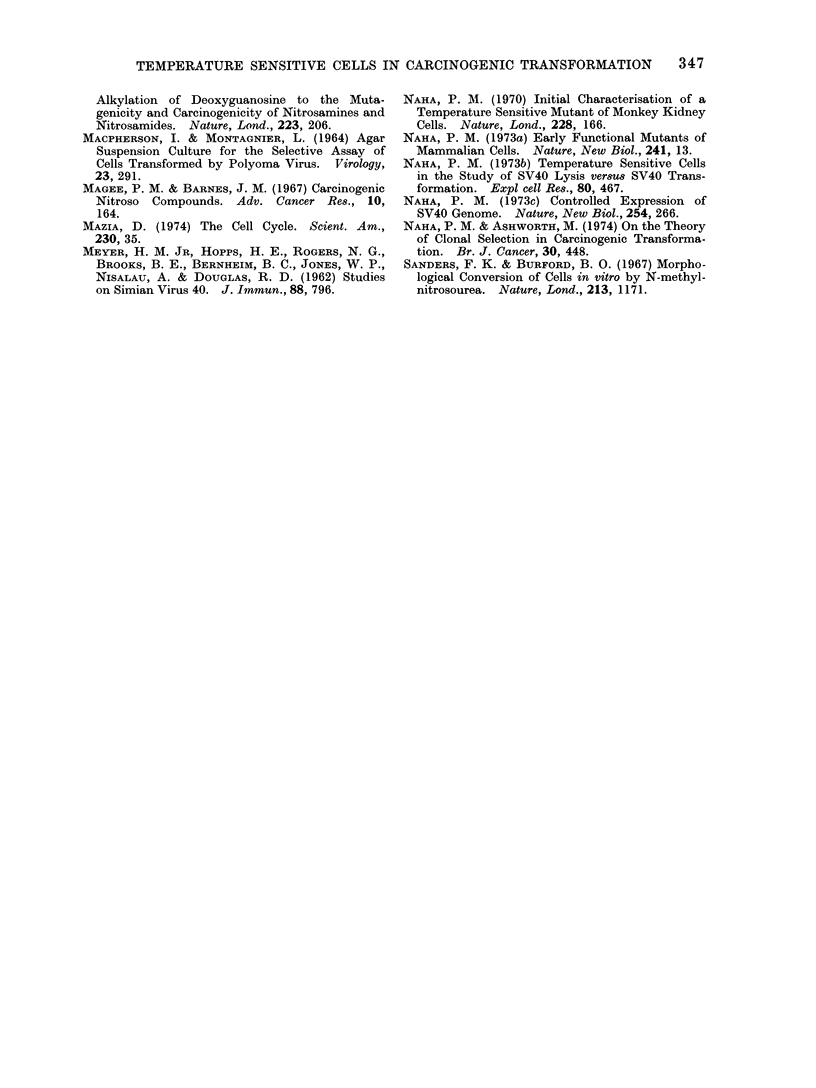# Temperature sensitive cells in the study of carcinogenic transformation.

**DOI:** 10.1038/bjc.1975.68

**Published:** 1975-03

**Authors:** P. M. Naha

## Abstract

**Images:**


*Br. J. Cancer* (1975) **31**, 338

### TEMPERATURE SENSITIVE CELLS IN THE STUDY OF CARCINOGENIC TRANSFORMATION

P. M. NAHA

*From the Paterson Laboratories, Christie Hospital and Ho t Radium Institute, Manchester M20 9BX, U.K.*

Received 18 November 1974. Accepted 10 December 1974

**Summary.—Two temperature sensitive variants (ts13 and ts14) of an African green monkey tetraploid kidney cell line (epithelial), carrying temperature sensitive lesions in thymidine metabolism, were transformed by methylnitrosourea (MNU) at the restricted temperature of 39·5°C; the same cell lines were not transformed by MNU at the permissive temperature of 33°C, nor was there any transformation in the wild type parental cell line of BSC-1 at these temperatures under similar conditions. A comparative study of the cell cycle and metabolic efficiency in the 3 cell lines was performed in order to get an understanding of the physiology of the "target cells" in culture. Compared with the parental cell line of BSC-1, ts13 and ts14 cells were blocked in the G1 phase of the cell cycle during the time that the cells were in contact with the carcinogen (MNU); the variant cells also had higher mitotic indices at this time. The cells of ts13 which showed 50% more transformation than those of ts14 differed from the latter in having larger numbers of viable cells arrested in mitosis over the G1 period. The results were interpreted to indicate that there were other factors, besides cells arrested being in G1, which contributed to the difference in the frequency of transformation between the variant cell lines which had an otherwise similar physiology. Using gel electrophoresis a new protein was located in the nuclei of the transformed cells of ts13 and ts14 which was absent in the wild type cell line of BSC-1 or in the variants ts13 and ts14 at 39·5°C.**

EXPERIMENTAL studies in carcinogenesis have relied almost exclusively on the success of inducing *in vitro* transformation in mammalian cells by chemical carcinogens (Berwald and Sachs, 1963; Heidelberger and Iype, 1967; Sanders and Burford, 1967) which opened the possibility of studying the mode of action of these carcinogens at the cellular and molecular level. We have described before (Naha and Ashworth, 1974) the use of a temperature sensitive variant cell line, carrying a biochemical lesion in thymidine metabolism, which showed a high frequency of transformation *in vitro,* induced by the carcinogen methylnitrosourea at the restricted temperature; the variant at the permissive temperature or the parental cell line showed no detectable change in cellular morphology under similar conditions. These experiments (Naha and Ashworth, 1974) suggested that transformation in the temperature-sensitive cell line was selective in nature and raised the possibility of a "clonal selection" in carcinogen transformation in mammalian cells, at least with respect to certain chemical carcinogens.

The induction of a "transformed state" in culture has been attributed (Heidelberger, 1973) to at least 2 cellular mechanisms: (1) direct transformation resulting from interaction of a chemical with some critical target in the cell or (2) activation of an oncogenic virus by the chemicals. There is a strong evidence from sero-epidermiological studies and from cell culture studies to support the hypothesis that cells of many vertebrates contain C-type RNA viruses, and partial expression of the viral genome by chemical and physical agents might be responsible for transforming the cell into a tumour cell (Huebner and Todaro, 1969). The "target cell" theory, on the other hand, has remained largely unsubstantiated because of poorly understood areas of interactions of chemicals with the cells (particularly in respect of mutagenesis versus carcinogenesis), and of the physiology of the "target cells" in culture. In this article the physiology of 2 temperature sensitive variant cell lines which differed in their susceptibility to transformation by methylnitrosourea are compared in order to understand the nature of the "target cells" in chemical carcinogenesis. In contrast to other chemical carcinogens, methylnitrosourea requires little enzymatic metabolism (Magee and Barnes, 1967). Cells treated only briefly with methylnitrosourea *in vitro* acquired a capacity for (morphologically) altered growth when cultured later, as well as an ability to grow as tumours in a heterologous host (Sanders and Burford, 1967).

### MATERIALS AND METHODS

*Cell lines*.—The cell lines used in these experiments were: the African green monkey SV40 sensitive tetraploid kidney (epithelial) cell line of BSC-1 (Meyer *et al.*, 1962) obtained from the Imperial Cancer Research Fund Laboratories, London, and its temperature-sensitive variants, ts13 and ts14 isolated by the methods described previously (Naha, 1973*a*). These cell lines grew as monolayers to confluence and were strongly contact inhibited in culture. Cells were maintained in stationary cultures at the permissive temperature (33 °C) in L-15 medium, supplemented with 10% foetal bovine serum (FBS), 100 u penicillin/ml and 50 $\mu$g streptomycin/ml, as described previously (Naha, 1970). The variant clones, ts13 and ts14, were non-producers of SV40 at the restricted temperature of 39·5 °C and were found to undergo transformation by SV40 at this temperature (Naha, 1973*b*); both these cell lines were lytic to the virus at the permissive temperature of 33 °C. Preliminary experiments (Naha, 1973*a*) indicated that the variant cell lines (reversion frequency $<10^{-4}$) were defective in the metabolism of exogenous thymidine, but thymidine triphosphates were synthesized in the precursor pools of both. The variant cell lines, however, differed in their ability to synthesize SV40 T (tumour) antigens (Naha, 1973*b*, *c*) at the restricted temperature though both were defective in the synthesis of V antigen. Whereas clone ts13 was positive for SV40 T antigen, clone ts14 was negative. It might be of interest to point out that the 2 variant clones were isolated from the same experiment under identical conditions of mutagenesis.

*Induction of transformation*.—Susceptibility to transformation in these cell lines was studied by the chemical carcinogen methylnitrosourea at concentrations below the levels of toxicity ($<100$ $\mu$g/ml). Very few transformants were observed above this level; the optimal concentration for inducing transformation was found to be about 40 $\mu$g/ml. The details of these studies have already been published (Naha and Ashworth, 1974). Cells at a density of $1 \times 10^5$ were plated in 25 cm$^2$ (30 ml) Falcon tissue culture flasks and incubated for 18 h (one cell generation time roughly 16–20 h) at the restricted temperature of 39·5 °C. The cultured cells were then washed in prewarmed Hanks' BSS (pH 6·8) for 15 min. N-methyl-N-nitrosourea (MNU), obtained from Dr A. W. Craig of the Carcinogenesis Unit of these laboratories, at the required concentrations in Hanks' BSS (pH 6·8) was added to the cultures and incubated for 2 h at 39·5 °C with appropriate controls. At the end of the treatment, MNU solutions were pipetted out and replaced with L-15 growth medium (Naha, 1970) and incubated at the permissive temperature of 33 °C for 2–4 weeks. Transformed cells (foci of uninhibited cells) began to appear in cultures of ts13 and ts14, but not in the parental culture of BSC-1 between 1 and 2 weeks after MNU treatment. Cloning of transformed cells from MNU treated cultures was found to be relatively easy. Lightly trypsinized cells in culture, when shaken vigorously, tended to release the clumped cells (foci) first and these were replated at suitable dilutions. These cultures grew as discrete colonies and the growth characteristics of these transformed clones will be reported elsewhere. The transformed clones were recorded as ts13/MNU and ts14/MNU. A number of transformed clones were isolated in each case.

*Transformation frequency*.—An improved method for scoring transformants was followed to compare the frequency of transformation in different cell lines induced by methylnitrosourea. Previously we described (Naha and Ashworth, 1974) a technique of counting under the microscope the number of foci per unit area on 2–3 week old cultures. This method was found to be unsatisfactory when making comparisons between different temperature sensitive cell lines, mainly because the number of viable cells at the end of the preincubation period (at the restricted temperature), and thus exposed to the carcinogen, was found to be different in different cell lines. In this improved method we accepted one selective technique from our previous experiments with these cell lines; the MNU treated transformed cells showed higher efficiency of plating at lower cell densities ($< 1 \times 10^4$ per flask). We thus trypsinized the MNU treated cells after overnight incubation at the permissive temperature of 33°C (described in the preceding section) and replated these cells at a density of 1000 viable cells/25 $cm^2$ Falcon tissue culture flasks at 33°C and 39·5°C. Replicate cultures of the same were put on soft agar (0·6% basal agar and 0·2% top agar) simultaneously. All plates were incubated for 3 weeks at their respective temperatures. Half of the medium in each flask was exchanged for fresh growth medium once every week, except for soft agar flasks which had an infusion of 1 ml of fresh growth medium once every week. Control cultures of (MNU) untreated cells and cells incubated at 33°C only (not exposed to restricted temperature) were treated in the same manner. The number of transformed foci was counted from Giemsa stained preparations at the end of the incubation period (3–4 weeks).

*Cell cycles*.—A comparative study of the cell cycles (Mazia, 1974) of BSC-1, ts13 and ts14 was performed at 33°C and 39·5°C in order to understand the phase in the cell cycle where the temperature sensitive cells were defective at the time of their exposure to MNU. In general, the method described by Baserga (1965) was followed. Cells at a density of $1 \times 10^5$ per ml were planted on coverslips in Leighton tubes in 1 ml volumes and incubated for 40 h at 33°C to allow the cells to reach the log phase of growth. Cells were then exposed to radioactive medium of thymidine-methyl-$^3$H, obtained from the Radiochemical Centre, Amersham, at a concentration of 3 $\mu$Ci/ml (3 Ci/mmol) for 10 min. The cells were then washed in Hanks' BSS containing 4 $\mu$g/ml of cold thymidine in order to dilute the remaining radioactivity and prevent further incorporation of radioactivity in the cells. Finally, the cells were placed in L-15 medium containing 4 $\mu$g/ml of cold thymidine; replicate tubes were incubated at 33°C and 39·5°C. At hourly intervals one set of tubes was removed, fixed and stained by the following method. After removing the medium the cells were treated with a hypotonic solution of Hanks' BSS in water (1 : 4) for 5 min, fixed for 5 min in acetic acid : ethanol (1 : 3) diluted in the hypotonic medium, and then fixed for a further period of 10 min in undiluted fixative. The coverslips were air dried and mounted in glass slides with the cells on the upper side. The slides were then dipped in Ilford L-4 liquid emulsion and left to expose for 2 weeks in the dark at −20°C. After 2 weeks the slides were developed in D19 developer for 4–5 min and fixed for 10 min in Fixol solution in water (1 : 4), washed for 1 h in running water. The slides were then completely dried before staining in buffered Giemsa (4%). The numbers of labelled cells in mitosis in 100 metaphase plates were counted for each sample. The mitotic index was calculated from the same preparations by counting the average number of cells in mitosis in 50 microscopic fields.

*DNA, RNA and protein synthesis*.—To correspond to the progress of the 3 lines (BSC-1, ts13 and ts14) through the critical stages of their cell cycle, it was thought necessary to record the physiology of these cells by studying their rates of incorporation of thymidine, uridine and leucine as an indication of their efficiency to metabolize DNA, RNA and protein. For this purpose, Leighton tubes with coverslips (same as above) were prepared; replicate cultures were exposed to thymidine-2-$^{14}$C (12·5 $\mu$Ci/ml, 5 Ci/mmol), uridine-5-$^3$H (28 $\mu$Ci/ml, 17·5 Ci/mmol) and L-leucine-$^{14}$C(G) (20$\mu$Ci/ml,

58 Ci/mmol) for 10 min in Hanks' BSS at 39·5°C. The coverslips were then washed twice with cold Hanks' BSS, treated with 10% cold trichloroacetic acid (TCA), washed in methanol and counted in toluene based scintillation fluid, as described before (Naha, 1970). Radioactive chemicals were obtained from the Radiochemical Centre, Amersham.

*Cell fusion.*—Cell fusion experiments were performed in the presence of (β-propiolactone) inactivated Sendai virus (Harris and Watkins, 1965) between ts13 and ts14 with appropriate controls. The clone ts22 was one of the early functional mutants (Naha, 1973*a*) which was blocked in DNA synthesis at the restricted temperature by way of defective uridine incorporation; this clone was used as a control to study effective complementation. For fusion, equal numbers of cells ($5 \times 10^4$) from each parent were mixed in presence of 800 HAU (Harris and Watkins, 1965) of inactivated Sendai virus. The controls of the individual parental cultures were plated at a cell density of $1 \times 10^5$ in the presence of the inactivated virus. Mixed cells and parental cultures were plated in 1 ml volumes in Leighton tubes containing coverslips as described above. Cell cultures were exposed to 30 μCi/ml (3 mCi/mmol) of thymidine-methyl-$^3$H for 20 min at 39·5°C. Cells were washed twice with cold Hanks' BSS, acid (TCA) and the precipitable fraction was counted as before (Naha, 1970).

*Analysis of the nuclear prcteins.*—Cloned cultures of BSC-1, ts13, ts13/MNU/4, ts14, ts14/MNU/2 were planted at a cell density of $1 \times 10^5$ per ml in 30 ml volumes in 16 oz glass bottles and incubated for 16 h at 39·5°C. Nuclear preparations and analysis of the nuclear proteins were made by the methods described previously (Naha, 1973*c*).

### RESULTS

### *Transformation frequency*

The improved technique of scoring of MNU induced transformation in these epithelial cell lines was found to have certain practical advantages. Though a particular selective condition (that of higher efficiency of plating at low cell density) was being imposed on the MNU treated cells by this method, isolation of transformants and quantitative analysis of transformation frequency between the two temperature sensitive cell lines were made much easier. Other alternative techniques attempted, as in the case of overlaying in soft agar, were relatively less successful and will be discussed later.

Table I shows the results of the experiments where cells were pre-incubated for 18 h at 39·5 C, then treated with MNU at concentrations of 0, 50 and 100 μg/ml, and finally plated at a density of 1000 viable cells/25 cm² Falcon tissue culture flask at 33°C and 39·5°C. Control experiments where BSC-1, ts13 and ts14 were incubated at 33°C only before being exposed to MNU did not produce any transformed colonies under otherwise similar experimental conditions.

The following observations were made from these experiments: (1) The inability to induce transformation by MNU in the wild type parental cell line of BSC-1 compared with ts13 and ts14 where MNU induced transformation was ob-

TABLE I.—*Frequency of Transformation Induced by Methylnitrosourea (MNU) in Cultures of BSC-1, ts13 and ts14; Experimental Conditions Described in the Text*

| Cell lines | MNU treatment (μg/ml) | Number of transformed* colonies per 1000 viable cells in 25 cm² area: 33°C | 39·5°C |
|---|---|---|---|
| BSC-1 | 0 | 0 | 0 |
| | 50 | 0 | 0 |
| | 100 | 0 | 0 |
| ts13 | 0 | 0 | 0 |
| | 50 | 98·8 | 23·5 |
| | 100 | 7·0 | 2·6 |
| ts14 | 0 | 0 | 0 |
| | 50 | 45·3 | 10·9 |
| | 100 | 4·2 | 1·0 |

* The discrete colonial, and piled up morphology was the most readily recognizable transformed property in these epithelial cell lines which were characterized by contact inhibited monolayer growth in culture.

served, indicated that transformation in the 2 temperature sensitive variant cell lines was somehow related to their temperature sensitive defects; (2) the frequency of transformation (induced by MNU at 50 $\mu$g/ml) was higher in ts13 (9·88%) than in ts14 (4·53); (3) comparing the number of transformants at 33°C and 39·5°C, it appeared that in both ts13 and ts14 at least one-fourth of the transformants were temperature insensitive (between these temperatures). Cloned cultures of temperature sensitive and temperature insensitive transformants are now being studied for other physiological changes. These findings support our previous observations (Naha and Ashworth, 1974) that transformation in these temperature sensitive variant cells was selective in nature and raised the possibility of "clonal selection" in carcinogenic transformation. It is possible that the temperature insensitive transformed clones arose because of a second mutation caused by MNU which was irrelevant to the incidence of transformation.

Replicate cultures on soft agar did not produce identical results; in both ts13 and ts14 the number of colonies growing on soft agar was 10 to 20 times fewer than their corresponding plastic tissue culture flasks. Even then, much smaller numbers of cells (only 8–10) per colony grew on soft agar during the period of 3 weeks, compared with plastic flasks where the number of cells per colony during this time reached 100–200. It is possible that the parameter of growth on soft agar as applicable to indicate transformation in fibroblast (Macpherson and Montagnier, 1964) does not altogether hold true for epithelial cells, or else that this property is not unique for all transformed cells or all forms of transformation.

*Cell cycle*

A comparative analysis of the cell cycles (Fig. 1A–1C) of BSC-1, ts13 and ts14 showed that though the 3 cell lines exhibited identical cell generation time at 33°C (about 20 h) and the cells progressed through different phases of the cell cycle at the same rate (duration of S phase was a little longer in ts13 and ts14) at this temperature, the variant cell lines ts13 and ts14 showed an "extreme damping" effect after the completion of the first cycles at the restricted temperature of 39·5°C. Compared with those at 33°C, the cell cycle time for BSC-1, ts13 and ts14 was much shorter for the first cycle at 39·5°C (about 14 h). Whereas the parental cell line of BSC-1 progressed normally into the second cycle at 39·5°C (Fig. 1A), the temperature sensitive variants ts13 (Fig. 1B) and ts14 (Fig. 1C) were held up in the G1 phase of the second cycle and did not enter into the S phase. The cause of this "damping" effect in ts13 and ts14 was not clear. It was evident from the results that both the variant cell lines had progressed to a certain extent into the G1 phase before being held up. It also appeared that both ts13 and ts14 proceeded along G1 to the same extent, after which the 2 variant cell lines differed in levelling off. Whereas ts13 showed a smooth plateau (Fig. 1B), the picture in ts14 was uneven (Fig. 1C). It was possible that a sub-population in ts14 was entering G1 phase at different times, or else the cells in ts13 were better synchronized at the end of the first cycle of 39·5°C. It should be mentioned here that the cells of ts13 and ts14 were exposed to the carcinogen (MNU) to induce transformation between 18 and 20 h at 39·5°C when the cells were arrested in G1 phase.

Analysis of the mitotic indices for these 3 cell lines at 39·5°C (Fig. 1A–1C) showed some remarkable differences. As expected, the parental cell line of BSC-1 appeared to be near normal, the mitotic index rising from 1·0% to 5·8% after 16 h of incubation at 39·5°C, after which it fell to 2·0% by 24 h (Fig. 1A). Compared with this, both ts13 and ts14 accumulated mitotic indices of up to

FIG. 1.—The cell cycle and mitotic indices of BSC-1 (Fig. A), ts13 (Fig. B) and ts14 (Fig. C) at 33°C and 39·5°C, and the rates of incorporation of radioactive thymidine, uridine and leucine at corresponding times by BSC-1 (Fig. D), ts13 (Fig. E) and ts14 (Fig. F). The viable cell counts at those times are presented in Fig. D–F.

8·2% and 8·4% respectively, after 16 h at 39·5°C. After this, the mitotic index in ts13 remained near 8·0% for the next 12 h (Fig. 2B), whereas that in ts14 fell slowly down to 2·0% after 30 h at 39·5°C (Fig. 1C). High mitotic indices in ts13 and ts14, compared with the parental BSC-1 cell line, were probably due to partial synchronization of cells because of the effects of restricted temperature; but this alone would not explain the high mitotic index in ts13 over such a long duration of time. The cells of ts13 were certainly not accumulating mitotic cells as in the case of colchicine treatment where one would notice an exponential increase in mitotic index. In this case there was a slow loss of rounded cells but otherwise the ratio of cells in different phases of mitosis had remained constant between 16 and 20 h without any significant difference (Table II). The ratio of cells in prophase, metaphase, anaphase and telophase was more or less similar between the 3 cell lines during the critical period when

TABLE II.—*Ratio of Mitotic Cells in Different Divisional Stages During* 16, 18 *and* 20 *h of Incubation at* 39·5°*C*

| | | Ratio in percentage | | | |
|---|---|---|---|---|---|
| Cell lines | Incubation time (h) | Pro-phase | Meta-phase | Ana-phase | Telo-phase |
| BSC-1 | 16 | 18·85 | 57·58 | 9·94 | 13·61 |
| | 18 | 19·30 | 54·33 | 10·07 | 16·30 |
| | 20 | 19·36 | 54·92 | 10·21 | 15·49 |
| ts13 | 16 | 12·00 | 58·17 | 10·64 | 19·18 |
| | 18 | 17·50 | 52·00 | 10·16 | 20·30 |
| | 20 | 12·22 | 61·90 | 10·47 | 15·23 |
| ts14 | 16 | 22·82 | 49·65 | 11·41 | 16·12 |
| | 18 | 23·10 | 45·53 | 15·53 | 16·34 |
| | 20 | 19·92 | 57·76 | 10·33 | 12·15 |

the cells were in contact with the carcinogen. The only difference between these cell lines was in the number of cells in mitosis at these times. To explain the situation in ts13 one could visualize that the entire process of mitosis was slowed down in this cell line at 39·5°C.

Viability counts of cells (Fig. 1D–1F) between 12 and 24 h showed that compared with the BSC-1 cell line, ts13 had 10% less viable cells at 39·5°C and ts14 had nearly 30% less. That would in effect imply that in ts13 and more truly in ts14 a fraction of cells going through mitosis might be irreversibly arrested in growth at the end of their division.

*Rates of DNA, RNA and protein synthesis*

A measurement of the rate of incorporation of radioactive thymidine, uridine and leucine between 10 and 26 h at 39·5°C was taken to note the general physiology of the cells of BSC-1, ts13 and ts14 in terms of their efficiency to synthesize DNA, RNA and protein, corresponding to the time when the cells were supposed to be entering their second cycle. These data indicated that in BSC-1 there was an increase in thymidine incorporation to correspond with the second S period without any detectable change in the rate of uridine and leucine uptake. Compared with this, thymidine incorporation in ts13 was slowed down considerably while that in ts14 actually fell. The restriction in thymidine incorporation probably accounted for the decrease in uridine and leucine incorporation in ts13; in ts14 absence of any noticeable thymidine uptake was immediately reflected in the fall in uridine and leucine incorporation, as also in the drop in viability counts. These data taken together were interpreted as indicating that compared with the BSC-1 cell line, ts13 cells were arrested in growth at 39·5°C, whereas the cells in ts14 were actually dying.

*Complementation between ts*13 *and ts*14

Cell fusion experiments with equal number of cells in a mixed population of ts13 and ts14, performed in the presence of inactivated Sendai virus, showed that there was poor complementation between these 2 cell lines (Fig. 2) when the cells approached the second S period of their cell cycle. While BSC-1 cells incorporated $^3$H-thymidine at a near exponential rate at this time, there was hardly any increase in ts13, ts14 or mixed cultures of ts13 and ts14 at 39·5°C. Another temperature sensitive cell line, ts22, which was blocked in DNA synthesis by way of defective uridine uptake in early G1 (Naha, 1973*a* and Naha, unpublished data) showed some degree of complementation with ts14 under the same conditions. The degree of complementation in the latter case also suggested that the temperature sensitive cells of ts13 and ts14 were probably non-complementing.

*Analysis of nuclear proteins*

Polyacrylamide gel electrophoretic studies of the nuclear proteins isolated from BSC-1, ts13, ts13/MNU/4, ts14 and ts14/MNU/2, performed in parallel cylindrical gels, showed (Fig. 3) the appearance of a unique protein in the MNU induced transformed cell lines of ts13/MNU/4

FIG 2.—Complementation tests (by cell fusion) between the temperature sensitive variants ts13, ts14 and ts22, measured by the rates of $^3$H-thymidine uptake, between ts13 and ts14, and ts22 and ts14, in the presence of inactivated sendai virus.

FIG. 3.—Polyacrylamide gel electrophoresis of 100 $\mu$g of nuclear proteins of the following cell cultures at 39·5°C: BSC-1 (a), ts13 (b), ts13/MNU/4 (c), ts14 (d), ts14/MNU/2 (e). Gels stained with 2% naphthalene black.

and ts14/MNU/2. This band of protein was absent from the parental cell lines of BSC-1, ts13 and ts14. The similar electrophoretic mobility of this new protein in ts13/MNU/4 and ts14/MNU/2 might be taken to indicate that the protein was identical in both cases. Because these 2 variant cell lines ts13 and ts14 were non-complementary and were blocked in the same phase (G1) of the cell cycle, it is tempting to suggest that the 2 transformed cell lines have arisen by the same process of cellular interaction with the carcinogen.

The morphological and biochemical characterization of the transformed clones (details to be published elsewhere) also showed remarkable similarities, though they were derived from two independent temperature sensitive variants. In general, the transformed clones were similar in (1) the loss of temperature sensitivity, (2) colony morphology, (3) high plating efficiency at low cell densities, (4) increased agglutination by concanavalin A and (5) tumorogenicity when injected into green monkeys (subcutaneously).

### DISCUSSION

The results presented in this paper demonstrate that temperature sensitive cells can be used successfully in experimental studies on chemical carcinogenesis, particularly with respect to cellular and molecular mechanisms. An improved method has been described for selection of transformed clones in epithelial cells induced by a carcinogen. The more important aspects of this study lie in quantitation of transformation frequency in the 2 variant cell lines which poorly complemented each other in thymidine uptake (but differed in certain aspects of cell physiology) and in the attempt to relate the difference in transformation frequency to the physiology of the " target cells " in culture.

The quantitative estimation of transformation frequency showed that the cell line ts13 had twice as many transformed cells as ts14 under similar conditions. Though an equal number of viable cells (1000 cells) were plated in culture after MNU treatment for the estimation of transformation frequency, the induction of transformation had already taken place during the period of 2 h that the cells were in contact with the carcinogen, probably by " single hit " kinetics. During this critical period (18–20 h of incubation at 39·5°C), when the cells were held up in G1 period of the cell cycle, the 2 variant cell lines differed in at least 2 aspects of their physiology: (1) high mitotic index (around 8%) in both, containing mostly viable cells in ts13 as opposed to fewer number of viable cells in ts14; (2) the cells of ts13 were still synthesizing DNA (as indicated by the rate of thymidine uptake), whereas the cells of ts14 were not. Which one of these 2 factors contributed to higher transformation frequency in ts13 was difficult to assess; it is probable that both these factors were necessary during methylation and stabilization of methylated DNA (Magee and Barnes, 1967; Loveless, 1969) as opposed to the process of repair during replication. By this argument the lower number of transformants in ts14 was probably due to the fact that at least some of the physiologically " target cells " had become non-viable and were excluded.

The relation of cell cycle to oncogenesis (Baserga, 1971) has been reported by other workers. Bertram and Heidelberger (1974) observed high frequency of transformation in mouse fibroblasts in starved (by amino acid deprivation) cultures arrested in G1. Our experiments also indicated that there might be a relationship between cells arrested in G1 and the frequency of transformation, but it is possible that at least in our case there were other contributory factors as well, *e.g.* a small population of cells held up in mitosis over G1 period. Such cells, when they are viable and capable of synthesizing DNA could form the " target cells " in carcinogenesis.

Location of a unique protein in the nuclei of the transformed cells of ts13/MNU/4 and ts14/MNU/2 during gel electrophoresis led us to believe that transformation in the 2 variant cell lines was caused by identical reactions. Whether this protein was in any way related to transformation is now being studied by careful scrutiny of the various revertants that were missing this protein.

It is of interest to point out that these 2 variant cell lines under identical conditions of arrested cell cycle also undergo transformation by SV40 (Naha, 1973*b*, *c*). It is indeed tempting to suggest that what we have been noticing is a convergence of viral and chemical oncogenesis. It would be remarkable if it turns out to be true.

This work was supported by grants from the Cancer Research Campaign and the Medical Research Council. The author acknowledges the excellent technical assistance provided by Mrs Kathleen Hewitt. Some of the early experiments on cell cycle were performed by Miss Margaret Ashworth and will be published in detail elsewhere.

REFERENCES

BASERGA, R. (1965) measurement of Cell Cycle by the Method of Labelled Mitoses. *Cancer Res.*, **25**, 581.

BASERGA, R. (1971) *The Cell Cycle and Cancer.* New York: Marcel Dekker Inc.

BERTRAM, J. S. & HEIDELBERGER, C. (1974) Cell Cycle Dependency of Oncogenic Transformation Induced by N-methyl-N′-nitro-N-nitrosoguanidine in Culture. *Cancer Res.*, **34**, 526.

BERWALD, Y. & SACHS, L. (1963) *In vitro* Cell Transformation with Chemical Carcinogens. *Nature, Lond.*, **200**, 1182.

HARRIS, H. & WATKINS, J. F. (1965) Hybrid Cells Derived from Mouse and Man: Artificial Heterokaryons of Mammalian Cells from Different Species. *Nature, Lond.*, **205**, 640.

HEIDELBERGER, C. (1973) Chemical Carcinogenesis in Culture. *Adv. Cancer Res.*, **18**, 317.

HEIDELBERGER, C. & IYPE, P. T. (1967) Malignant Transformation *in vitro* by Carcinogenic Hydrocarbons. *Science, N.Y.*, **155**, 214.

HUEBNER, R. J. & TODARO, G. J. (1969) Oncogenes of RNA Tumor Viruses as Determinants of Cancer. *Proc. natn. Acad. Sci. U.S.A.*, **64**, 1087.

LOVELESS, A. (1969) Possible Relevance of O-6

Alkylation of Deoxyguanosine to the Mutagenicity and Carcinogenicity of Nitrosamines and Nitrosamides. *Nature, Lond.*, **223**, 206.

MACPHERSON, I. & MONTAGNIER, L. (1964) Agar Suspension Culture for the Selective Assay of Cells Transformed by Polyoma Virus. *Virology*, **23**, 291.

MAGEE, P. M. & BARNES, J. M. (1967) Carcinogenic Nitroso Compounds. *Adv. Cancer Res.*, **10**, 164.

MAZIA, D. (1974) The Cell Cycle. *Scient. Am.*, **230**, 35.

MEYER, H. M. JR, HOPPS, H. E., ROGERS, N. G., BROOKS, B. E., BERNHEIM, B. C., JONES, W. P., NISALAU, A. & DOUGLAS, R. D. (1962) Studies on Simian Virus 40. *J. Immun.*, **88**, 796.

NAHA, P. M. (1970) Initial Characterisation of a Temperature Sensitive Mutant of Monkey Kidney Cells. *Nature, Lond.*, **228**, 166.

NAHA, P. M. (1973*a*) Early Functional Mutants of Mammalian Cells. *Nature, New Biol.*, **241**, 13.

NAHA, P. M. (1973*b*) Temperature Sensitive Cells in the Study of SV40 Lysis *versus* SV40 Transformation. *Expl cell Res.*, **80**, 467.

NAHA, P. M. (1973*c*) Controlled Expression of SV40 Genome. *Nature, New Biol.*, **254**, 266.

NAHA, P. M. & ASHWORTH, M. (1974) On the Theory of Clonal Selection in Carcinogenic Transformation. *Br. J. Cancer*, **30**, 448.

SANDERS, F. K. & BURFORD, B. O. (1967) Morphological Conversion of Cells *in vitro* by N-methylnitrosourea. *Nature, Lond.*, **213**, 1171.